# *Pediococcus acidilactici* FZU106 alleviates high-fat diet-induced lipid metabolism disorder in association with the modulation of intestinal microbiota in hyperlipidemic rats

**DOI:** 10.1016/j.crfs.2022.04.009

**Published:** 2022-04-27

**Authors:** Qing Zhang, Wei-Ling Guo, Gui-Mei Chen, Min Qian, Jin-Zhi Han, Xu-Cong Lv, Li-Jiao Chen, Ping-Fan Rao, Lian-Zhong Ai, Li Ni

**Affiliations:** a Institute of Food Science and Technology, College of Biological Science and Engineering, Fuzhou University, Fuzhou, Fujian, 350108, PR China; b College of Food Science, Fujian Agriculture and Forestry University, Fuzhou, 350002, PR China; c Food Nutrition and Health Research Center, School of Advanced Manufacturing, Fuzhou University, Jinjiang, Fujian, 362200, PR China; d School of Medical Instruments and Food Engineering, University of Shanghai for Science and Technology, Shanghai, 200093, PR China; e College of Light Industry and Food Science, Zhongkai University of Agriculture and Engineering, Guangzhou, Guangdong, 510225, PR China; f International Joint Research Center for Probiotics & Gut Health, School of Food Science and Technology, Jiangnan University, Wuxi, Jiangsu, 214122, PR China

**Keywords:** *Pediococcus acidilactici* FZU106, Hyperlipidemia, Lipid metabolism, Intestinal microbiota, mRNA expression

## Abstract

Probiotics have been proved to have beneficial effects in improving hyperlipidemia. The purpose of the current research was to investigate the ameliorative effects of *Pediococcus acidilactici* FZU106, isolated from the traditional brewing of *Hongqu* rice wine, on lipid metabolism and intestinal microbiota in high-fat diet (HFD)-induced hyperlipidemic rats. Results showed that *P. acidilactici* FZU106 intervention obviously inhibited the abnormal increase of body weight, ameliorated serum and liver biochemical parameters related to lipid metabolism and oxidative stress. Histopathological evaluation also showed that *P. acidilactici* FZU106 could significantly reduce the excessive lipid accumulation in liver caused by HFD-feeding. Furthermore, *P. acidilactici* FZU106 intervention significantly increased the short-chain fatty acids (SCFAs) levels in HFD-fed rats, which was closely related to the changes of intestinal microbial composition and metabolism. Intestinal microbiota profiling by high-throughput sequencing demonstrated that *P. acidilactici* FZU106 intervention evidently increased the proportion of *Butyricicoccus*, *Pediococcus*, *Rothia*, *Globicatella* and *[Eubacterium]_coprostanoligenes*_group, and decreased the proportion of *Corynebacterium*_1, *Psychrobacter*, *Oscillospira*, *Facklamia*, *Pseudogracilibacillus*, *Clostridium_innocuum*_group, *Enteractinococcus* and *Erysipelothrix* in HFD-fed rats. Additionally, *P. acidilactici* FZU106 significantly regulated the mRNA levels of liver genes (including *CD36*, *CYP7A1*, *SREBP-1c*, *BSEP*, *LDLr* and *HMGCR*) involved in lipid metabolism and bile acid homeostasis. Therefore, these findings support the possibility that *P. acidilactici* FZU106 has the potential to reduce the disturbance of lipid metabolism by regulating intestinal microflora and liver gene expression profiles.

## Highlights

• *Pediococcus acidilactici* FZU106 protects against hyperlipidemia.
• *Pediococcus acidilactici* FZU106 regulates serum and liver lipid levels.
• *Pediococcus acidilactici* FZU106 regulates intestinal microbial composition.
• *Pediococcus acidilactici* FZU106 regulates lipid metabolism related genes.

## Graphical abstract

**Figure:**
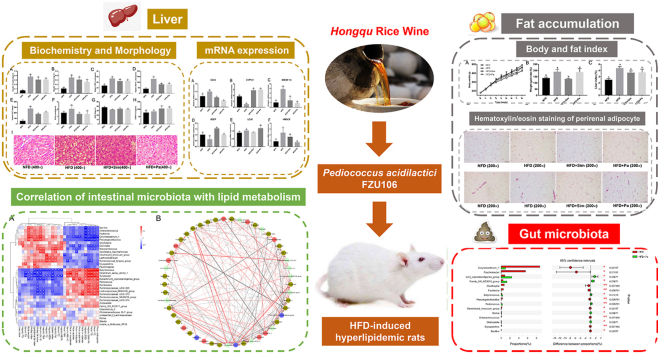

## 1 Introduction

Over the past ten years, the incidence rate of metabolic syndromes (MetS) caused by excessive high-fat diet (HFD) has been increasing sharply worldwide, becoming a major public health problem and clinical challenge (Ishikawa et al., 2020; Morris et al., 2015). MetS is mainly characterized by metabolic abnormalities, including diabetes, hyperglycemia, atherosclerosis, hyperlipidemia and fatty liver, seriously affecting one quarter of adults around the world. Hyperlipidemia, a typical metabolic disease mainly featured by abnormally increase of cholesterol and triglyceride concentrations in blood and liver, is closely related to the increased fatality rate of cardiovascular diseases (CVDs), affecting about millions of people around the world (Ren et al., 2021). In recent years, the prevalence of hyperlipidemia in people of different gender and age has increased sharply all over the world (Tang et al., 2019). At present, drug therapy is considered to be the main treatment for hyperlipidemia, while most lipid-lowering drugs have a series of unpleasant side effects (Liu et al., 2018; Zhang et al., 2020a). Looking for natural products and probiotics from food resources with strong ameliorative effects on lipid metabolism is a promising strategy to intervene the pathological development of hyperlipidemia.

Probiotics are defined as live microbial cells that bring potential health benefits to the host when administered in adequate quantities (Yoo and Kim, 2016; Yeon-Jeong et al., 2014). Oral probiotics may ameliorate hyperlipidemia through regulating the composition and metabolic function of intestinal flora (He and Shi, 2017; Jiang et al., 2020). Among the probiotics used commercially, lactic acid bacteria (LAB) are dominant representatives of probiotics that play a positive role in promoting human health, and mainly used in the production of various fermented foods (Peters et al., 2019; Wuyts et al., 2020). Accumulating evidences have shown that daily consumption of LAB and LAB fermented foods can significantly improve or prevent fatty liver, hypercholesterolemia, hyperlipidemia and even cancer (Fasseas et al., 2013; Saadat et al., 2019). In rodent models of obesity induced by high-fat diet, the anti-obesity and anti-hyperlipidemic effects of *Lactobacillus*, *Bifidobacterium* and *Pediococcus* have been widely reported by many researchers (Wang et al., 2020). *Pediococcus acidilactici*, belonging to the family Lactobacillaceae, is recognized as a safe probiotic by the European Food Safety Authority, and commonly used as starter culture in the fermentation of fruit and vegetable because it can inhibit the growth of spoilage microorganisms (Irmler et al., 2013). There is increasing evidence that the consumption of probiotic *P. acidilactici* is a potentially effective way for triglycerides and cholesterol-lowering (Jain et al., 2013; Ooi and Liong, 2010). For instance, *P. acidilactici* FS2 isolated from Korean traditional fermented seafood showed high resistance to acids and bile, and this strain can significantly reduce blood cholesterol level when taken with isomalto-oligosaccharides (Jang et al., 2021). *P. acidilactici* M76 strain isolated from Korean traditional makgeolli presented excellent lipid-lowering effects on obese mice induced by high-fat diet through suppressing the key lipid synthesis enzymes. Wang et al. (2019) demonstrated that oral administration of *P. acidilactici* AS185 from traditional farmers' soybean paste alleviated the development of atherosclerosis by regulating lipid metabolism and preventing inflammation. Our previous preliminary experiment showed that *P. acidilactici* FZU106 isolated from the traditional fermentation of *Hongqu* rice wine was a potential probiotic strain with bile tolerance and cholesterol-lowering ability (Supplementary material-Tables S1 and S2). However, there is lack of study about the *in vivo* protective effects of *P. acidilactici* FZU106 against hyperlipidemia.

Intestinal microbiota has been widely recognized as one of the most important determinants affecting host lipid metabolism (Lv et al., 2019). Previous study indicated that the occurrence of hyperlipidemia is usually accompanied by the disorder of intestinal microbiota (Villanueva-Millán et al., 2015). It is well known that probiotics regulate the ecological balance of intestinal microbiota and produce antibacterial substances (such as bacteriocins) and competing with pathogenic bacteria for adhesion and colonization in the intestine, so as to prevent or alleviate the occurrence of metabolic syndrome (Kim et al., 2019). Among the commonly used probiotics, many strains belonging to *Lactobacillus* and *Pediococcus* have been preliminarily proved to have the effect of improving or intervening hyperlipidemia (Chen et al., 2020a; Oh et al., 2019; Lv et al., 2021). *L. plantarum* PMO 08 administration significantly inhibited fat accumulation, enhanced intestinal integrity and regulated the composition of intestinal bacteria (Oh et al., 2019). *L. plantarum* HNU082 intervention markedly enhanced the levels of *Bifidobacterium*, *Lactobacillus*, *Akkermansia* and *Faecalibacterium* in hyperlipidemic rats (Shao et al., 2017). *L. paracasei* FZU103 administration has the potential to protect against non-alcoholic fatty liver related to hyperlipidemia (Lv et al., 2021). *P. pentosaceus* PP04 isolated from traditional pickled cabbage has been proved to improve hyperlipidemia induced by high-fat diet by regulating lipid metabolism (Wang et al., 2020). Dietary supplementation of probiotics *P. acidilactici* MA18/5M regulated intestinal flora and stimulated various non-specific immune parameters in zebrafish (Jaramillo-Torres et al., 2019). Random clinical trials have shown that *P. acidilactici* promoted immunity to activate IgA production, and improve the intestinal flora and intestinal function (Kim et al., 2018; Fernandez et al., 2015). However, the improvement effect of *P. acidilactici* administration on the intestinal microbial composition under high-fat diet and its association with lipid metabolism need to be further explored.

This research tried to investigate the potential benefits of *P. acidilactici* FZU106 on lipid metabolism in hyperlipidemic rats. Whether *P. acidilactici* FZU106 intake can alter the intestinal microbiota composition was also investigated through 16S amplicon high-throughput sequencing, and microbial metabolism functions were predicted. Besides, the connections between the key microbial phylotypes in intestinal tract and the lipid metabolism were revealed through correlation analysis and visualized by network.

## 2 Materials and methods

### 2.1 Probiotic strain and culture conditions

*P. acidilactici* FZU106, isolated from the traditional brewing process of *Hongqu* rice wine, was offered by Institute of Food Science and Technology, Fuzhou University. The original strain of *P. acidilactici* FZU106 was frozen in −80 °C refrigerator. During the experiment, the original strain of *P. acidilactici* FZU106 was thawed and activated in Man-Rogosa-Sharpe (MRS) broth (at 37 °C for 24 h) every three days. After three generations, cells of live cultures were washed twice with sterile saline, and then re-suspended in sterile saline (adjusted to 1.0 × 10^9^ CFU/mL) for oral administration in animal experiment.

### 2.2 Animals and experimental protocols

Forty male Sprague-Dawley (SD) rats (6-week-old) were purchased from Shanghai Laboratory Animal Center (Shanghai, China), and housed in a SPF grade animal laboratory (temperature: 24 ± 1 °C, relative humidity: 60 ± 5%, light-dark cycle: 12 h), the Animal Center of Institute of Food Science and Technology, Fuzhou University. All rats were given free access to diet and water. After one week of acclimatization, all rats were randomly divided into four groups as follow: (1) NFD group (n = 8, fed a standard chow diet and oral gavage with 1.0 mL sterile physiological saline per day); (2) HFD group (n = 8, fed a high-fat diet and oral gavage with 1.0 mL sterile physiological saline per day); (3) HFD + Sim group (n = 8, fed a high-fat diet and oral gavage with simvastatin [20 mg/kg per day]); (4) HFD + Pa group (n = 8, fed a high-fat diet and oral gavage with cells of *P. acidilactic* [10^9^ CFU/rat per day]). The compositions of the standard chow diet and high-fat diet (composing 30% fat energy-supply ratio) were shown in Supplementary Table S1. The body weight of each rat was recorded every week in the experimental period. The animal experimental protocols were conducted in accordance with the guidelines of the Laboratory Animal Welfare and approved by the Animal Ethics Committee of Institute of Food Science and Technology, Fuzhou University, China (No.: FZU-FST-2021-066).

### 2.3 Sample collection and biochemical assays

After eight weeks’ intervention, fecal samples from each rat were collected in the frozen tubes in short time and then stored in the −80 °C refrigerator for the quantification of fecal short-chain fatty acids (SCFAs). All rats were starved for 12 h and then euthanized under anesthesia with 2% sodium pentobarbital. Blood were collected into tubes and centrifuged at 3000 rpm for 10 min at 4 °C to obtain serum samples that were stored at refrigerator (−80 °C) until further analysis. Liver samples were dissected immediately, weighted, frozen in liquid nitrogen, and finally maintained at an ultra-cold freezer (−80 °C) until further analysis. Cecal content samples from each dissected rat were collected in the frozen tubes in short time and then stored in the −80 °C refrigerator for high throughput sequencing.

Total cholesterol (TC), triglycerides (TG), low-density lipoprotein cholesterol (LDL-C), high-density lipoprotein cholesterol (HDL-C), total bile acid (TBA), malondialdehyde (MDA), glutathione peroxidase (GSH-Px), superoxide dismutase (SOD) and non-esterified fatty acids (NEFAs) were quantified using assay kits (Nanjing Jiancheng, Nanjing, China) according to the manufacturer's instructions. The total protein contents in liver samples were measured using a bicinchoninic acid (BCA) protein assay kit and quantified according to the manufacturer's instructions (Beyotime Biotec. Co., Ltd., Shanghai, China).

### 2.4 Hematoxylin–eosin (H&E) staining

For histopathological evaluation, fresh liver sections were excised and washed with cold physiologic saline (0.9%), and then fixed in 4% paraformaldehyde solution for 24 h, following by dehydration through a series of ethanol solutions, and embedded in paraffin and cut into section (5 μm thickness). After the fabricated liver sections were viewed using a light microscope (Olympus, Tokyo, Japan) equipped with a digital camera.

### 2.5 Determination of the total lipid levels in liver

The hepatic total lipids were extracted based on a previously published method with some modifications. Total lipids in the livers were extracted with chloromethane (CHCl_3_)/methanol (2 : 1, v/v) and incubated at 4 °C for 24 h. Then, the mixtures were dispersed in 0.6% KCl solution and centrifuged at 2000 g for 20 min. The organic layer was concentrated and solubilized in 200 μL of isopropanol and measured using commercial kits (Labtest Diagnostica S.A., MG, Brazil).

### 2.6 Quantification of fecal short-chain fatty acids (SCFAs)

The SCFAs in fecal samples were extracted and determined according to our previously reported method with appropriate modifications (Guo et al., 2020). Briefly, saturated NaCl solution (500 μL) was added to dried feces (50 mg) and placed at room temperature (25 °C) for 0.5 h, followed by homogenization on a high-speed homogenizer for 3 min. Then, 20 μL H_2_SO_4_ (10%, v/v) was added and mixed with a vortex for 30 s. The total SCFAs were collected with 800 μL anhydrous ether and then centrifuged (10000 g, 10 min, 4 °C). Finally, the residual trace water in the supernatants were removed with anhydrous Na_2_SO_4_, and the contents of SCFAs in the supernatants were determined by Agilent 7890B gas chromatography system equipped with Agilent J&W DB-WAX capillary column (30 m × 0.25 mm × 0.25 μm) and flame ionization detector.

### 2.7 High throughput sequencing and bioinformatics analysis

Bacterial DNAs were extracted from cecal content samples using genomic DNA extraction kit (Omega, USA) and quantified by NanoDrop Spectrophotometer (Thermo Fisher Scientific). The V3–V4 hypervariable regions of bacterial 16S rDNA sequence were amplified using the primers 338F and 806R, and then sequenced by high throughput sequencing based on Illumina MiSeq platform at Shanghai Majorbio Co., Ltd. (Shanghai, China). The raw data from high throughput sequencing that support the findings of this study are openly available at https://www.ncbi.nlm.nih.gov/genbank (Reference number: PRJNA812963).

The sequencing raw data were imported into QIIME 2 software, and the filtered sequences were clustered into operation taxon units (OTU) with 97% identity threshold. Based on the GreenGenes database (Ver. 13.8), the sequence similarity was matched to identify microbial phylotypes at the genus level, and the relative abundance of each OTU was obtained. Principal component analysis (PCA) and hierarchical clustering were implemented to assessed the intestinal microbial composition by SIMCA (Ver. 15.0). The different taxonomies between different groups were revealed at the genus level using STAMP software (Ver. 2.1.3). The differences between groups were determined using Welsh's *t*-test, and the Benjamini-Hochberg procedure was used to control the false-discovery rate. The microbial functional features of the intestinal microbiota were predicted using PICRUSt 2.0 based on the relative abundance of the identified microbial phylotypes. Correlation heatmap between the intestinal microbial phylotypes and the lipid metabolic parameters was drawn using R software (Ver. 3.3.3). Correlation network between the key intestinal microbial phylotypes and lipid metabolic parameters was visualized using Cytoscape software (Ver. 3.6.0).

### 2.8 Reverse transcription-quantitative polymerase chain reaction (RT-qPCR)

Total hepatic RNA was extracted by a commercial RNA extraction kit (RNAiso Plus, Code No. 9108) provided by Takara Biomedical Technology (Beijing, China) Co., Ltd., and then reverse-transcribed into cDNA using a commercial cDNA kit with gDNA Eraser [Code No. RR047A] (Takara, Beijing, China). qPCR was completed in StepOne Plus Real-Time quantitative PCR System (Applied Biosystems, Foster City, CA, USA) with SYBE Green Ex Taq™ II [Tli RNaseH Plus, Code No. RR820A] (Takara, Beijing, China). The PCR conditions were as following: initial activation 95 °C for 30s, denaturation 95 °C for 5s, annealing 55 °C for 30 s, extension 72 °C for 30 s, 40 cycles. The mRNA expressions level was normalized to *β-Actin* gene. In this study, the 2^−ΔΔCT^ method was used to analyze the relative expression levels of related genes. The qPCR primers used in this study were purchased from Shanghai Sangon Biotech. Co., Ltd. (Shanghai, China) (Table 1).

**Table 1:** Primer sequence for quantitative real-time PCR.

Gene	Forward primer (5′-3′)	Reverse primer (5′-3′)
*LDLr*	TGGCTATGAGTGCCTATGTCC	GGTGAAGAGCAGAAACCCTATG
*BSEP*	CGTGCTTGTGGAAGAAGTTG	GGGAGTAGATGGGTGTGACTG
*HMGCR*	AGTGGTGCGTCTTCCTCG	CGAATCTGCTGGTGCTAT
*CD36*	GACAATCAAAAGGGAAGTTG	CCTCTCTGTTTAACCTTGAT
*SREBP-1c*	GCTGTTGGCATCCTGCTATC	TAGCTGGAAGTGACGGTGGT
*CYP7A1*	CACCATTCCTGCAACCTTTT	GTACCGGCAGGTCATTCAGT
*β-Actin*	ACGTCGACATCCGCAAAGACCTC	TGATCTCCTTCTGCATCCGGTCA

### 2.9 Statistical analysis

All values are expressed as mean ± SEM. The significance of differences among the experimental group were evaluated by one-way analysis of variance (ANOVA) using GraphPad Prism (Ver. 6.0), followed by Tuckey's multiple-comparison test. When *P* value was less than 0.05, the difference was considered statistically significant.

## 3 Results

### 3.1 Effects of *P. acidilactici* FZU106 intervention on body growth performance

**Fig. 1:**
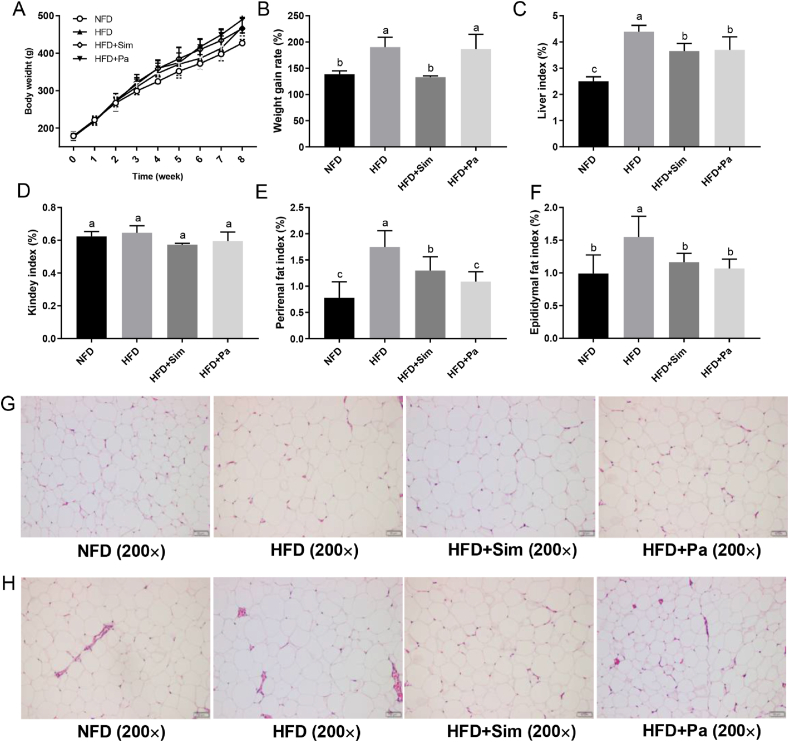
Effects of *Pediococcus acidilactici* FZU106 administration on the body growth performance and fat accumulation. (A) Body weight growth; (B) body weight gain; (C) liver index; (D) Kidney index; (E) perirenal fat index; (F) epididymal fat index; (G) histopathological observation of perirenal adipocytes and (H) epididymal adipocytes from rats of different experimental groups by H&E staining at 100× magnification and 200× magnification. Values were expressed as mean ± SEM (n = 8), and different letters represent significant differences between different experimental groups (*P* < 0.05).

As shown in Fig. 1A, there was no significant difference in the initial body weight of rats among different experimental groups at the beginning of the experiment. However, HFD-feeding for eight weeks induced a significant growth in body weight compared with the NFD group. This undesirable growth trend caused by HFD-feeding was effectively alleviated by the daily intervention of *P. acidilactici* FZU106 (Fig. 1A&B). Notably, HFD-fed rats also displayed significant increases in the indices of liver, perirenal and epididymal adipocytes, when compared with the NFD-fed rats (*P* < 0.05) (Fig. 1C–F). *P. acidilactici* FZU106 intervention significantly prevented the abnormal growth of liver, perirenal and epididymal adipocytes caused by HFD (*P* < 0.05). Subsequently, the microstructures of perirenal and epididymal adipocytes were also illustrated in all experimental groups (Fig. 1G&H). The volumes of perirenal and epididymal adipocytes were smaller in the rats of the HFD + Sim and HFD + Pa groups, compared with those of the HFD group (Fig. 1G&H), indicating that daily intervention with *P. acidilactici* FZU106 may attenuated the swelling of adipocytes induced by HFD-feeding.

### 3.2 Effects of *P. acidilactici* FZU106 intervention on serum biochemical parameters

**Fig. 2:**
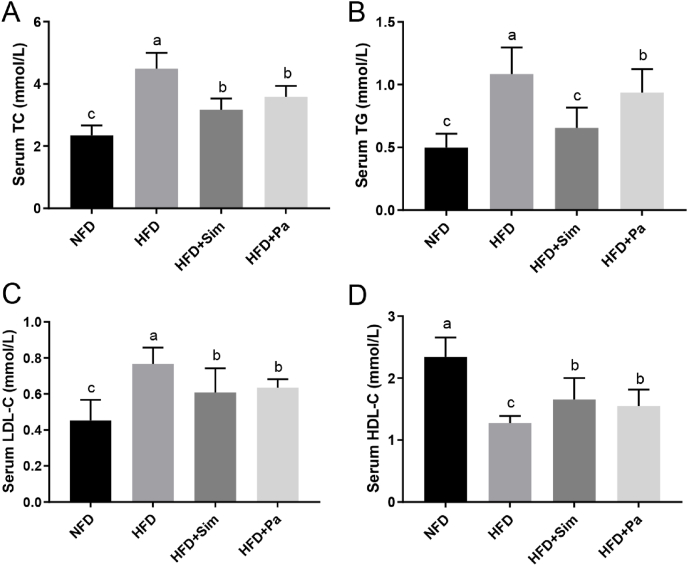
Effects of *Pediococcus acidilactici* FZU106 administration on the serum (A) TC; (B) TG, (C) LDL-C and (D) HDL-C levels in rats fed a high-fat diet. Values were expressed as mean ± SEM (n = 8), and different letters represent significant differences between different experimental groups (*P* < 0.05).

After 8 weeks’ experiment, the serum levels of TC, TG and LDL-C were sharply increased in the HFD-fed rats in comparison with the NFD group (*P* < 0.05) (Fig. 2). However, oral administration of *P. acidilactici* FZU106 obviously reduced the serum TG, TC and LDL-C levels and improved the serum HDL-C level in HFD-induced hyperlipidemic rats (*P* < 0.05), indicating that *P. acidilactici* FZU106 has a significant hypolipidemic effect.

### 3.3 Effects of *P. acidilactici* FZU106 intervention on liver biochemical parameters and histological features

**Fig. 3:**
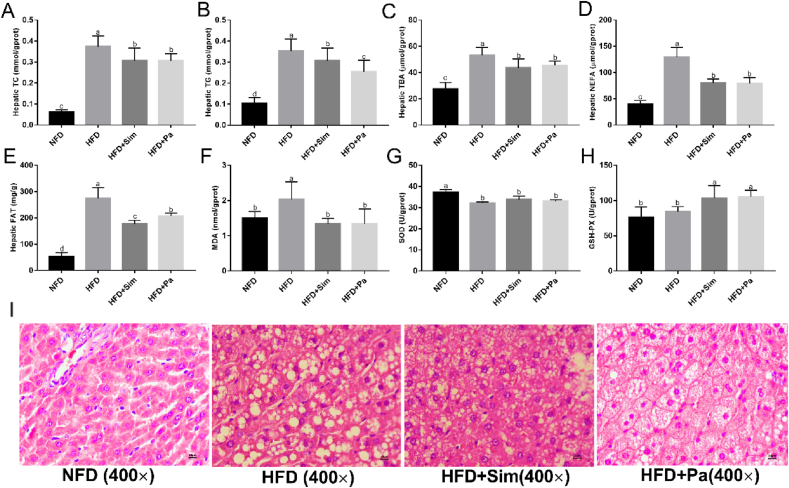
Effects of *Pediococcus acidilactici* FZU106 administration on hepatic lipid profile in HFD-fed rats. (A) Hepatic total cholesterol (TC), (B) total triglyceride (TG); (C) total bile acids (TBA); (D) non-esterified fatty acids (NEFA); (E) fat content; (F) MDA content; (G) SOD activity; (H) GSH-Px activity; (I) liver histopathological features (magnification × 400) by H&E staining. Values were expressed as mean ± SEM (n = 8), and different letters represent significant differences between different experimental groups (*P* < 0.05).

To evaluate the effect of *P. acidilactici* FZU106 intervention on liver lipid metabolism, hepatic biochemical parameters related to lipid metabolism and oxidative stress were determined by biochemical test kits. As shown in Fig. 3A–E, the HFD-induced hyperlipidemic rats were characterized by higher levels of TC, TG, TBA, NEFA and fat in the livers, compared with those of the NFD group (*P* < 0.05). Simvastatin intervention significantly prevented these adverse changes in the HFD-fed rats. Similar to simvastatin, the HFD-fed rats treated with *P. acidilactici* FZU106 showed lower levels of hepatic TC, TG, and NEFA compared with the HFD group. Biochemical analysis of lipid content in the liver confirmed this result. In addition, the effects of *P. acidilactici* FZU106 intervention on hepatic oxidative stress in HFD-fed rats were illustrated in Fig. 3F–H, and result showed that oral administration of *P. acidilactici* FZU106 sharply reduced the hepatic MDA level in HFD-fed rats (*P* < 0.05), but significantly increased the hepatic GSH-Px activity (*P* < 0.05), suggesting that *P. acidilactici* FZU106 intervention could alleviate liver oxidative damage by elevating the activities of antioxidant enzymes and inhibiting the oxidative stress in liver in hyperlipidemic rats induced by HFD-feeding. Histological analysis by H&E staining showed that the livers of the HFD-induced hyperlipidemic rats were characterized by excessive accumulation of lipid droplets (Fig. 3I). However, the size and number of lipid droplets in the HFD + Pa group were smaller and less than the HFD-induced hyperlipidemic rats, suggesting that the abnormal accumulation of liver lipids induced by HFD-feeding may be alleviated by *P. acidilactici* FZU106 intervention.

### 3.4 Effects of *P. acidilactici* FZU106 intervention on fecal biochemical parameters

**Fig. 4:**
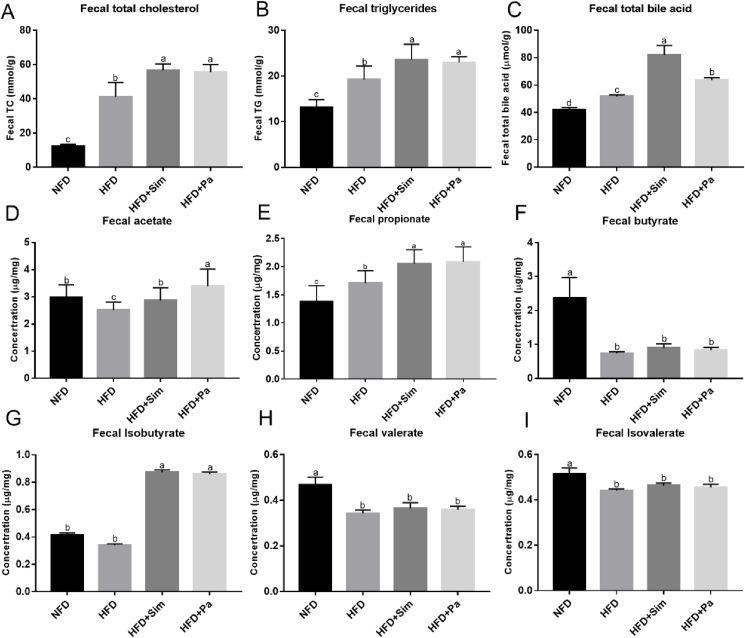
Effect of *Pediococcus acidilactici* FZU106 administration on the fecal lipid levels in rats fed a high-fat diet. (A) Fecal TC; (B) TG; (C) TBA, and short-chain fatty acids (SCFAs) including (D) acetate; (E) propionate; (F) n-butyrate; (G) isobutyrate; (H) valerate and (I) isovalerate. Values were expressed as mean ± SEM (n = 8), and different letters represent significant differences between different experimental groups (*P* < 0.05).

Results showed that oral administration of *P. acidilactici* FZU106 significantly upregulated the contents of fecal TC, TG and TBA compared with the HFD group (*P* < 0.05) (Fig. 4A–C), illustrating that *P. acidilactici* FZU106 intervention could effectively accelerate the excretion of fecal lipid (TC, TG, and TBA) through the intestinal tract. Short chain fatty acids (SCFAs), play an important role in health, mainly come from the breakdown of polysaccharide and dietary fiber are fermented to generate and are beneficial to human health. Compared with the rats of the HFD group, *P. acidilactici* FZU106 intervention significantly increased the fecal levels of acetate, propionate and isobutyrate (*P* < 0.05) (Fig. 4D–I).

### 3.5 Effects of *P. acidilactici* FZU106 intervention on intestinal microflora composition

**Fig. 5:**
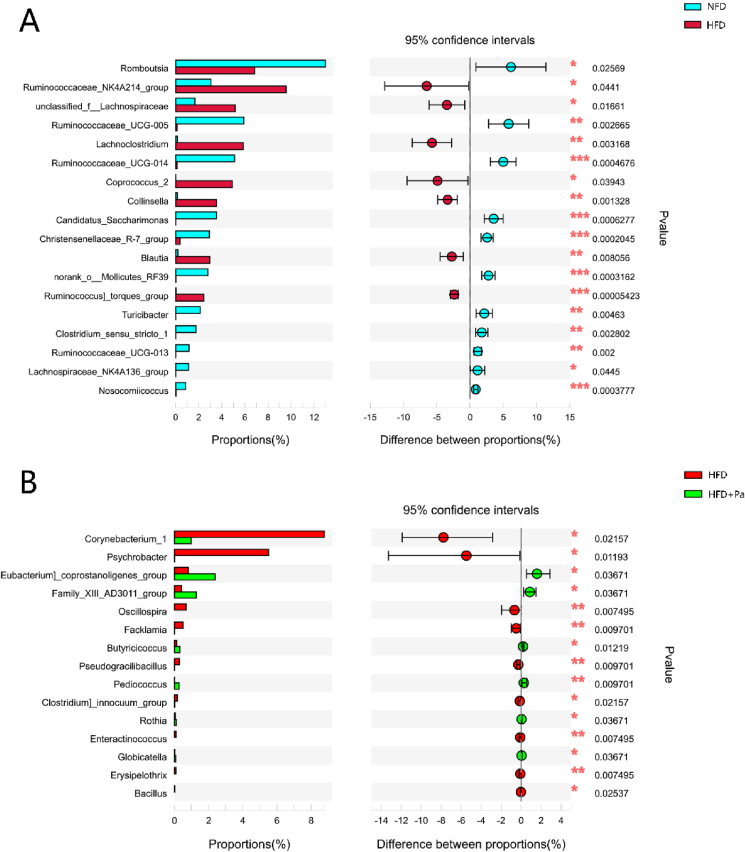
Extended error bar plot comparing the differences in the mean proportions of significantly altered genera. Benjamini-Hochberg procedure was used to control the false-discovery rate due to multiple testing. Corrected *P* values are shown at right. (A) The NFD group *versus* the HFD group; (B) the HFD group *versus* the HFD + Pa group. The confidence intervals are provided to allow for critical assessment of the biological relevancy of the test results.

Taxon-based analysis at the genus level revealed specific bacterial phylotypes among different experimental groups, including the NFD, HFD and HFD + Pa groups (Fig. 5). As compared with the NFD group, the HFD-induced hyperlipidemic rats (rats of the HFD group) presented higher levels of *Ruminococcaceae*_NK4A214_group, unclassified_f_*Lachnospiraceae*, *Lachnoclostridium*, *Coprococcus*_2, *Collinsella*, *Blautia*, *Ruminococcus]_torques*_group, but lower relative abundance of *Romboutsia*, *Ruminococcaceae_*UCG_005, *Ruminococcaceae_*UCG_014, *Candidatus_Saccharimonas*, *Christensenellaceae*_R-7_group, norank_o_Mollicutes_RF39, *Turicibacter*, *Clostridium_sensu_stricto*_1, *Ruminococcaceae_*UCG-013, *Lachnospiraceae*_NK4A136_group and *Nosocomiicoccus* at genus level, indicating that intestinal microbial dysbiosis occurred in the HFD-induced hyperlipidemic rats. However, oral administration with *P. acidilactici* FZU106 significantly increased the proportion of *Eubacterium]_coprostanoligenes*_group, Family_XIII_AD3011_group, *Butyricicoccus*, *Pediococcus*, *Rothia*, *Globicatella*, but decreased the proportion of *Corynebacterium*_1, *Psychrobacter*, *Oscillospira*, *Facklamia*, *Pseudogracilibacillus*, *Clostridium]_innocuum*_group, *Enteractinococcus*, *Erysipelothrix*, *Bacillus* in HFD-fed rats.

### 3.6 Metabolic function prediction of the intestinal microbiota by PICRUSt

**Fig. 6:**
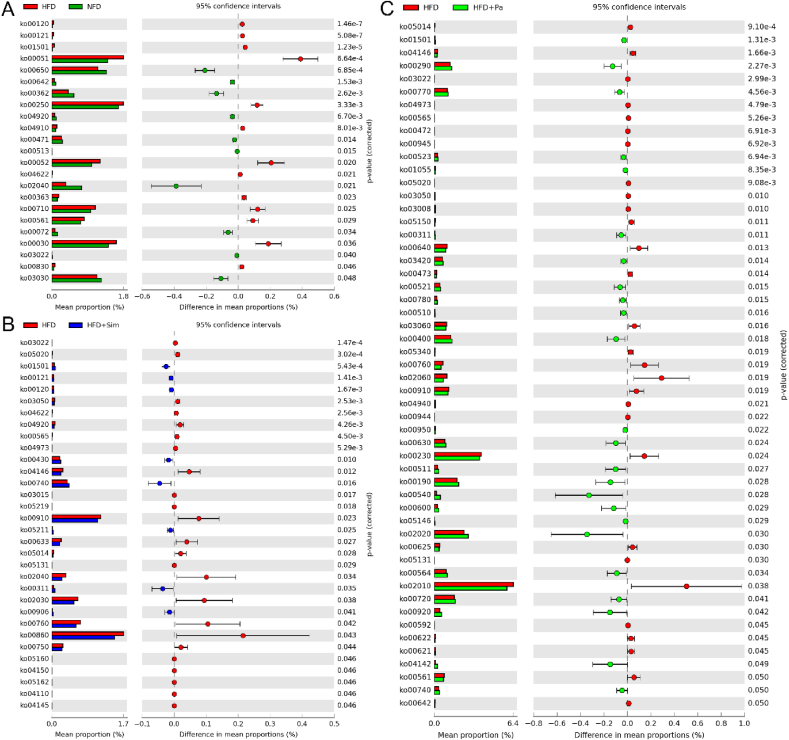
Extended error bar plot comparing the differences of significantly altered metabolic functions of intestinal microbiota by PICRUSt 2. The rightmost is the *P*-value, and *P* < 0.05 was regarded as statistically significant.

To explore the beneficial effects of *P. acidilactici* FZU106 intervention on the metabolic function of intestinal microbiota in rats fed with HFD, PICRUSt analysis based on 16S rRNA gene sequences and KEGG database was performed to predict the relationship between phylogeny and metabolism (Fig. 6). Compared with the HFD group, penicillin and cephalosporin biosynthesis (ko00311), phenylalanine, tyrosine and tryptophan biosynthesis (ko00400), oxidative phosphorylation (ko00190), lipopolysaccharide biosynthesis (ko00540), sphingolipid metabolism (ko00600), glycerophospholipid metabolism (ko00564) were enriched in the HFD-fed rats treated with *P. acidilactici* FZU106. On the contrary, ether lipid metabolism (ko00565), D-arginine and D-ornithine metabolism (ko00472), stilbenoid, diarylheptanoid and gingerol biosynthesis (ko00945), propanoate metabolism (ko00640), D-alanine metabolism (ko00473), purine metabolism (ko00230), glycerolipid metabolism (ko00561) were significantly down-regulated in the HFD-fed rats treated with *P. acidilactici* FZU106 compared with the HFD-induced hyperlipidemic rats.

### 3.7 Correlation of intestinal microbiota with HFD-induced lipid metabolic disorders

**Fig. 7:**
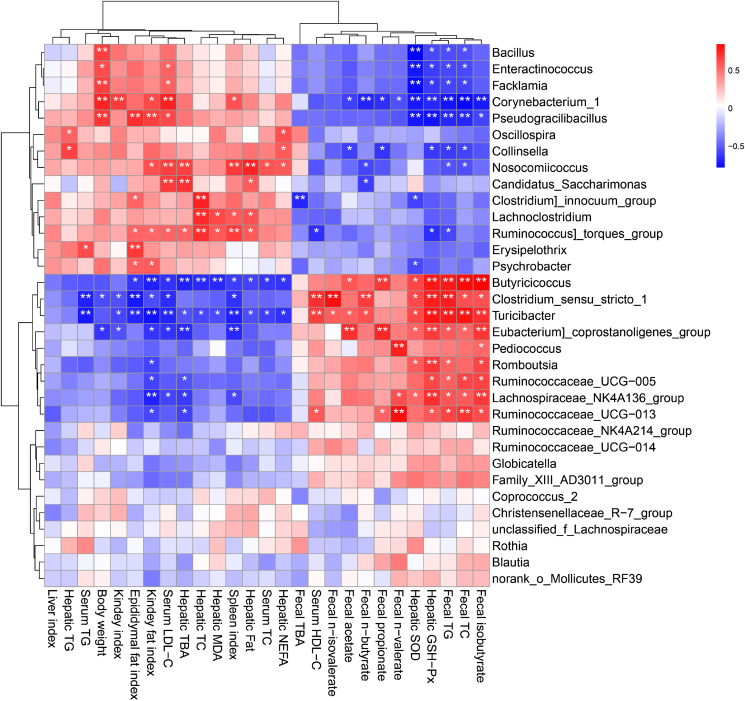
Statistical spearman's correlations between the fecal microbiota of significant differences and lipid metabolic parameters.

Correlation between the key microbial phylotypes and lipid metabolic disorders related biochemical parameters was conducted by Spearman's correlation analysis at the genus level (Fig. 7 and Fig. S1). Correlation heatmap and network analysis showed that the serum and hepatic lipid metabolism related biochemical parameters were positively correlated with *Corynebacterium*_1, *Pseudogracilibacillus*, *Facklamia* and *Nosocomiicoccus*, but negatively correlated with *Butyricicoccus*, *Pediococcus*, *Turicibacter*, *Clostridium_sensu_stricto_*1, *[Eubacterium]_ coprostanoligenes*_ group and *Lachnospiraceae*_NK4A136_group. Specifically, body weight showed a negative correlation with *Clostridium_sensu_stricto_*1 and *[Eubacterium]_ coprostanoligenes*_ group, but a positive correlation with *Corynebacterium*_1, *Facklamia*, *Pseudogracilibacillus*, *Enteractinococcus* and *Bacillus*. In addition, the serum TC, TG, LDL-C levels and hepatic TC, TG, NEFA levels were positively correlated with *Nosocomiicoccus*, *Corynebacterium*_1, *Pseudogracilibacillus* and *Oscillospira*, but showed significantly negative correlations with *Butyricicoccus*, *Pediococcus*, *Turicibacter*, *Clostridium_sensu_stricto_*1, *Lachnospiraceae*_NK4A136_group, *Ruminococcaceae_*UCG-013, and *[Eubacterium]_coprostanoligenes*_group. It is clear that fecal SCFAs (including acetic acid, propionic acid, n-butyric acid, isobutyric acid, n-valeric acid, isovaleric acid) were correlated positively with *Butyricicoccus Clostridium_sensu_stricto_*1, *Turicibacter*, *[Eubacterium]_coprostanoligenes*_group, *Pediococcus*, *Lachnospiraceae*_NK4A136_group and *Ruminococcaceae_*UCG-013.

### 3.8 Effects of *P. acidilactici* FZU106 intervention on liver mRNA levels of lipid metabolism-related genes

**Fig. 8:**
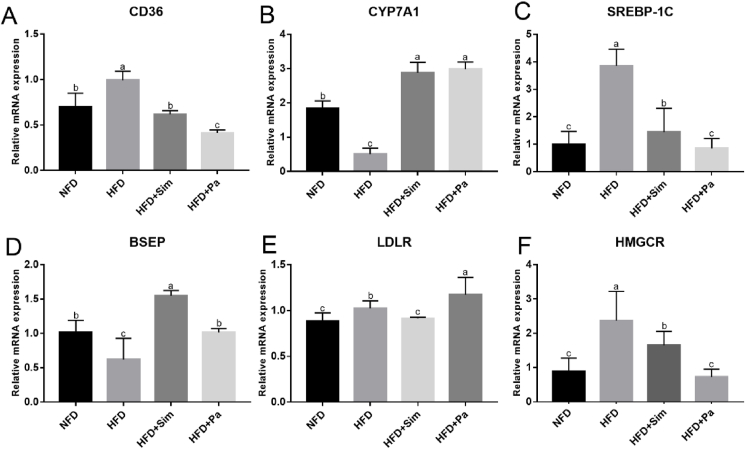
Effects of FLJ consumption on the expression of hepatic related genes in HFD-fed rats. The bar graphs showed the mRNA levels of (A) *CD36*, (B) *CYP7A1*, (C) *SREBP-1C*, (D) *BSEP*, (E) LDLR, and (F) *HMGCR*, which were determined by RT-qPCR. Values were expressed as mean ± SEM in each group (n = 8), and the different letters represent significant differences between different groups (*P* < 0.05).

Liver is an important organ involving in energy metabolism, fatty acid metabolism, and bile acid biosynthesis. To elucidate the mechanism underlying the hypolipidemic effects of *P. acidilactici* FZU106 intervention, the mRNA levels of genes responsible for cholesterol metabolism and bile acids homeostasis in liver were determined by real-time quantitative PCR (RT-qPCR) in the present study. As indicated in Fig. 8, eight-week HFD diet lowered hepatic mRNA levels of cholesterol 7α-hydroxylase (*CYP7A1*) and bile salt export pump (*BSEP*), compared with rats of the NFD group, while oral administration of *P. acidilactici* FZU106 promoted the mRNA expression of *CYP7A1*, *BSEP* and *LDLr* in liver. The hepatic mRNA levels of cluster of differentiation 36 (*CD36*), sterol regulatory element-binding protein-1c (*SREBP-1c*), low density lipoprotein receptor (*LDLr*) and 3-hydroxy-3-methylglutaryl-CoA reductase (*HMGCR*) were significantly higher in the HFD-induced hyperlipidemic rats than those of the NFD group. Nevertheless, *P. acidilactici* FZU106 intervention significantly decreased the mRNA levels of *CD36*, *SREBP-1c* and *HMGCR*, compared with the HFD-induced hyperlipidemic rats.

## 4 Discussion

The present study indicated that dietary oral administration of *P. acidilactici* FZU106 effectively prevented HFD-induced hyperlipidemia, ameliorated liver metabolism function and intestinal microbiota composition. Mounting evidences suggest that intestinal microbes play an important role in the pathological development of hyperlipidemia and liver metabolism function (Lv et al., 2019; Villanueva-Millán et al., 2015; Kim et al., 2019). In addition, previous study showed that intestinal flora is closely associated with lipid absorption in the intestine (Martinez-Guryn et al., 2018). Recent study by Zhu et al. (2021) revealed that fecal microbiota transplantation (FMT) regulates lipid absorption in the intestinal tract by altering the intestinal microbiota composition, which greatly affects the relative abundance of *Lactobacillus* and *Romboutsia* in the intestine. It is reported that *Lactobacillus* and *Romboutsia* are involved in maintaining intestinal epithelial barrier function, and their metabolites regulate lipid metabolism, thereby improving HFD-induced hyperlipidemia (Li et al., 2019; Russell et al., 2019; Yang et al., 2020). Based on the genus level analysis, we found that excessive consumption of high-fat diet induced significant reductions in the abundance of beneficial bacterial phylotypes, especially SCFAs-producing bacteria, including *Romboutsia*, *Turicibacter*, *Ruminococcaceae_*UCG_005, *Ruminococcaceae_*UCG-013, *Lachnospiraceae*_NK4A136_group, and *Clostridium_sensu_stricto*_1 (Xie et al., 2017; Mao et al., 2012; Zhong et al., 2015; Lanjekar et al., 2015). The genus Romboutsia was regarded as a typical bacterium whose abundance was positively correlated with serum HDL-c and SOD levels, but negatively correlated with serum MDA level based on correlation analysis (Li et al., 2021; Zheng et al., 2020). *Ruminococcaceae* and *Lachnospiraceae* are generally considered to promote intestinal health because they can anaerobically ferment carbohydrates into SCFA, especially acetic acid and propionic acid (Qu et al., 2017; Ghaly et al., 2020). Additionally, some genera of *Ruminococcaceae* produce acetate, which is subsequently metabolized by *Roseburia* to synthetize butyrate (Chen et al., 2020b). Early study had shown that the genus of *Lachnospiraceae* can inhibit the occurrence and development of liver cirrhosis and also improve atherosclerotic lesions (Ge et al., 2020). The genus Turicibacter was regarded as a typical bacterium whose abundance was positively correlated serum HDL-c and SOD levels, and might play a role in inflammatory bowel diseases (Gerritsen et al., 2019; Zheng et al., 2020).

Interestingly, our current research revealed that *P. acidilactici* FZU106 intervention ameliorated the intestinal microbiota dysbiosis by significant enhancing the relative abundance of *Butyricicoccus*, *Pediococcus*, *Rothia*, *Globicatella*, *Eubacterium_coprostanoligenes*_group and Family_XIII_AD3011_group, but significant reducing the proportion of *Corynebacterium*_1, *Psychrobacter*, *Oscillospira*, *Facklamia*, *Pseudogracilibacillus*, *Clostridium_innocuum*_group, *Enteractinococcus*, *Erysipelothrix* and *Bacillus* at the genus levels, which may be closely associated with the effective regulation of lipid metabolism in HFD-fed rats. As one of the putative SCFAs-producing bacteria, *Butyricicoccus* was previously reported to be negatively correlated with lipid metabolic disorders (Shang et al., 2017) and beneficial for liver and intestinal health, because butyric acid produced by *Butyricicoccus* provides lower intestinal pH, reduces the growth of harmful intestinal bacteria, and therefore prevents intestinal dysfunction (Zhang et al., 2015; Geirnaert et al., 2014). In this study, the relative abundance of *Butyricicoccus* was negatively correlated with fecal butyric acid level, suggesting that it may be closely related to the improvement of lipid metabolism. In recent decades, the function and role of *Pediococcus* in regulating lipid metabolism has received considerable attention (Wang et al., 2020). Many strains belonging to the genus *Pediococcus* have been proved to regulate lipid metabolism in *vivo*. *P. pentosaceus* LP28 displayed an anti-obesity effect by down-regulation of the serum levels of TG, TC, and LDL-C in human clinical trials (Higashikawa et al., 2016). *P. acidilactici* M76 and *P. pentosaceus* KID7 presented outstanding lipid-lowering and cholesterol-lowering effects in obese mice, respectively (Moon et al., 2014; Damodharan et al., 2015). In this study, the relative abundance of *Pediococcus* was negatively associated with the serum TC, TG and LDL-C levels, indicating that *Pediococcus* may be beneficial for reducing the risk of hyperlipidemia. In addition, excess cholesterol in blood comes from endogenous synthesis mainly in the liver and the small intestine. *[Eubacterium]_coprostanoligenes*_group, a cholesterol-reducing anaerobe in feces, has been found decreased in the HFD-induced hyperlipidemic rats and could generate beneficial SCFAs and have beneficial effects on dyslipidemia (Freier et al., 1994; Wan et al., 2019; Si et al., 2018). Previous study had shown that *[Eubacterium]_coprostanoligenes*_group can decompose cholesterol into sterols that cannot be absorbed in the intestine and is finally excreted with feces (Yang et al., 2020b). In addition, *[Eubacterium]_coprostanoligenes*_group is considered to be the pivotal genus in the fecal microecosystem mediating the effect of HFD on dyslipidemia through sphingosine (Wei et al., 2021). Metabolic function prediction of the intestinal microbiota by PICRUSt revealed that *P. acidilactici* FZU106 intervention significantly up-regulated phenylalanine, tyrosine and tryptophan biosynthesis and glycerophospholipid metabolism. Previous study indicated that the microbial degradation of aromatic amino acids (tryptophan, tyrosine and phenylalanine) into phenylacetic acid was significantly enhanced in patients with liver steatosis (Hoyles, L., Fernández-Real, JM., Federici, M. et al., 2018). It was previously reported that abnormalities in amino acid metabolism are closely associated with lipid metabolism disorder and CVDs (Shearer et al., 2008). Phenylalanine and tyrosine are precursors of epinephrine, which is required for lipid metabolism (Zhang et al., 2009). Tyrosine can be converted into fumaric acid and acetoacetate after a series of metabolic reactions, and then participates in the tricarboxylic acid cycle. Dysregulation of glycerophospholipid metabolism is considered as one of the main driving factors of lipid metabolism disorders, and is closely related to the development of T2DM and CVDs (Meikle and Summers, 2017). Therefore, *P. acidilactici* FZU106 intervention alleviates HFD-induced lipid metabolism disorder may be related to its regulation of phenylalanine, tyrosine and tryptophan metabolism, increased tyrosine production and reduced phenylalanine amounts. These results indicated that oral administration of *P. acidilactici* FZU106 is expected to play a hypolipidemic effect by regulating the intestinal microbiota. Of course, the metabolism of intestinal microbiome needs to be further explored by metabolomics technology based on LC-MS and GC-MS.

Liver plays a key role in maintaining lipid metabolism homeostasis (Fei et al., 2017; Huang et al., 2020). Thus, reducing liver lipid accumulation can efficiently inhibit the occurrence of hyperlipidemia. The results of this study showed that HFD-feeding led to excessive lipid accumulation in hyperlipidemic rats, which is consistent with previous study (Cheng et al., 2017). Interestingly, oral supplementation of *P. acidilactici* FZU106 efficiently increased the hepatic cholesterol level and promoted fecal excretion of intestinal bile acids (BAs) in HFD-induced hyperlipidemic rats. Cholesterol in the liver is usually processed to synthesize primary BAs, which are conjugated to glycine or taurine and stored in the gallbladder, and timely released into the intestine for solubilizing and emulsifying lipids in the intestine (Yamasaki et al., 2020). It has been reported that most of BAs could be reabsorbed by small intestine and will enter the liver and recycled, and the remaining BAs are excreted through the feces (Hubbard et al., 2006). Interestingly, the primary BAs can be converted into secondary BAs by microbial modification in the gut. Therefore, the improvement of lipid metabolism disorders in the body by probiotics is probably achieved by regulating the composition of intestinal microbiota and its metabolic function, thus promoting the biosynthesis and transformation of BAs (Chen et al., 2019). To elucidate the potential mechanism of action of *P. acidilactici* FZU106 intervention on lipid metabolism, the liver mRNA levels of key genes (including *HMGCR*, *CYP7A1*, *BSEP*, *CD36*, *SREBP-1c* and *LDLr*) related to lipid and cholesterol synthesis, as well as bile acid homeostasis, were measured through RT-qPCR. Our results showed that oral administration of *P. acidilactici* FZU106 made significant up-regulations on mRNA levels of *HMGCR* and *CYP7A1*, the two rate-limiting enzymes for cholesterol synthesis and cholesterol degradation, respectively (Ma et al., 2017). Especially, the up-regulation of *CYP7A*1 mRNA level may be the molecular mechanism of *P. acidilactici* FZU106to reduce cholesterol level in this study (Li et al., 2010). In addition to the biosynthesis of BAs, *BSEP* is the main hepatic membrane transporter involved in BAs excretion (Sultana et al., 2021). It can be seen that HFD-feeding significantly reduced the transcription level of liver *BSEP* gene. However, oral administration of *P. acidilactici* FZU106 can enhance the liver expression of *BSEP* gene to a certain extent. Moreover, we found that *P. acidilactici* FZU106 intervention could also significantly decrease the expression of hepatic genes of *CD36*, *SREBP-1c*, and *LDLr*, compared with the HFD group. Expression levels of these genes in the liver are upregulated in NAFLD model, suggesting a crucial role of lipid synthesis in hepatic steatosis (Fu et al., 2018; Zhang et al., 2020bb). As a key gene responsible for regulating lipid uptake in hepatocytes, the expression level of *CD36* is closely related to the pathological process of fatty liver and insulin resistance (Nergiz-Unal et al., 2020). Previous study had shown that *CD36* transports fatty acids into cells through *FATP*, thereby promoting the formation and accumulation of intracellular triglycerides (Nergiz-Unal et al., 2020). In this study, *P. acidilactici* FZU106 intervention prevented the uptake of fatty acids by decreasing the mRNA level of *CD36* in HFD-fed rats. In addition, *SREBP-1c* can regulate the transcriptional level of genes encoding enzymes involved in the synthesis and uptake of cholesterol and fatty acids in hepatocytes (He et al., 2016). Previous study had revealed that long-term dietary supplementation with probiotics can significantly reduce the liver mRNA level of *SREBP-1c*, which is a key lipogenic transcription factor that directly regulates the expression of rate-limiting enzymes of lipid synthesis and enzymes related to lipid uptake, thereby improving lipid metabolism (Oh et al., 2019). In additions, *P. acidilactici* FZU106 may also act on genes related to other than lipid metabolism pathway in the liver. Therefore, the expression and regulation of global genes in the liver need to be systematically analyzed by transcriptomics and proteomics.

Oxidative stress in liver has been thought to play a key role in lipid metabolism disorder, hyperlipidemia and NAFLD (Wang et al., 2012). In the present study, the lipid peroxidation indicator MDA in liver tissue was increased significantly in the HFD-fed rats, whereas the activities of SOD in liver tissue was decreased significantly. NAFLD patients have both elevated production of reactive oxygen species (ROS) and lowered antioxidant capacity (Suleiman et al., 2020). Liver lipid peroxidation would further lead to the production of some harmful by-products, such as MDA and ROS, which could activate inflammatory response and lead to hepatocyte damage (Takahashi and Mori, 2011; Uzun et al., 2009). In the present study, rats with *P. acidilactici* FZU106 intervention exhibited decreased MDA level and enhanced GSH-Px activity in liver. Previous report also demonstrated that *P. acidilactici* intervention inhibited lipid peroxidation in ANIT-induced cholestasis and in hepatic ischemia/reperfusion injury (Wang et al., 2014). The potential effect of *P. acidilactici* FZU106 in preventing liver oxidative stress may be achieved by reducing free radical production or through increasing free radical scavenging activity, which may be closely related to the metabolites of intestinal microbes.

## 5 Conclusion

This current study provided the evidence that *P. acidilactici* FZU106 consumption has the potential to alleviate hyperlipidemia in HFD-induced hyperlipidemic rats. The beneficial effects of *P. acidilactici* FZU106 consumption may be achieved by regulating intestinal flora and liver gene involved in lipid metabolism and bile acid homeostasis. The findings of this study have preliminarily revealed that *P. acidilactici* FZU106 can improve lipid metabolism disorder by regulating intestinal microflora and metabonomic profile. In further study, the protective mechanisms of *P. acidilactici* FZU106 against hyperlipidemia need to be clarified through liver transcriptomics, proteomics and metabonomics, as well as clinical crowd trials combined with multi-omics technology, so as to provide more credible references for the development of a promising functional food to prevent hyperlipidemia, and ultimately to benefit human health.

## CRediT authorship contribution statement

**Qing Zhang:** Investigation, Writing – original draft. **Wei-Ling Guo:** Investigation, Writing – original draft. **Gui-Mei Chen:** Investigation, Data curation. **Min Qian:** Funding acquisition, Formal analysis, Investigation, Software. **Jin-Zhi Han:** Investigation, Data curation. **Xu-Cong Lv:** Conceptualization, Funding acquisition, Supervision, Writing – review & editing. **Li-Jiao Chen:** Supervision, Methodology. **Ping-Fan Rao:** Supervision, Methodology, Resources. **Lian-Zhong Ai:** Resources, Conceptualization. **Li Ni:** Methodology, Validation, Project administration, Writing – review & editing.

## Declaration of competing interest

The authors declare that they have no known competing financial interests or personal relationships that could have appeared to influence the work reported in this paper.

## References

[bib1] Chen Y.J., Zhang J., Zhu P.P., Tan X.W., Lin Q.H., Wang W.X., Yin S.S., Gao L.Z., Su M.M., Liu C.X., Xu L., Jia W., Sevrioukova I.F., Lan K. (2019). Stereoselective oxidation kinetics of deoxycholate in recombinant and microsomal CYP3A enzymes: deoxycholate 19-hydroxylation is an in vitro marker of CYP3A7 activity. Drug Metab. Dispos..

[bib2] Chen M., Guo W.L., Li Q.Y., Xu J., Cao Y.J., Liu B., Yu X.D., Rao P.F., Ni L., Lv X.C. (2020). The protective mechanism of *Lactobacillus plantarum* FZU3013 against non-alcoholic fatty liver associated with hyperlipidemia in mice fed a high-fat diet. Food Funct..

[bib3] Chen M., Hou P., Zhou M., Ren Q., Wang X., Huang L., Hui S., Yi L., Mi M. (2020). Resveratrol attenuates high-fat diet-induced non-alcoholic steatohepatitis by maintaining gut barrier integrity and inhibiting gut inflammation through regulation of the endocannabinoid system. Clin. Nutr..

[bib4] Cheng H.S., Ton S.H., Phang S., Tan J., Kadir K.A. (2017). Increased susceptibility of post-weaning rats on high-fat diet to metabolic syndrome. J. Adv. Res..

[bib5] Damodharan K., Lee Y.S., Palaniyandi S., Yang S.H., Suh J. (2015). Preliminary probiotic and technological characterization of *Pediococcus pentosaceus* strain KID7 and in vivo assessment of its cholesterol-lowering activity. Front. Microbiol..

[bib6] Fasseas M.K., Fasseas C., Mountzouris K.C., Syntichaki P. (2013). Effects of *Lactobacillus salivarius, Lactobacillus reuteri*, and *Pediococcus acidilactici* on the nematode *Caenorhabditis elegans* include possible antitumor activity. Appl. Microbiol. Biotechnol..

[bib7] Fei T.L., Lim S.M., Ramasamy K. (2017). Cholesterol lowering by pediococcus acidilactici lab4 and *Lactobacillus plantarum* lab12 in adult zebrafish is associated with improved memory and involves an interplay between npc1l1 and abca1. Food Funct..

[bib8] Fernandez B., Savard P., Fliss I. (2015). Survival and metabolic activity of pediocin producer *Pediococcus acidilactici* UL5: its impact on intestinal microbiota and *Listeria monocytogenes* in a model of the human terminal ileum. Microb. Ecol..

[bib9] Freier T.A., Beitz D.C., Li L., Hartman P.A. (1994). Characterization of *Eubacterium coprostanoligenes* sp. nov. a cholesterol-reducing anaerobe. Int. J. Syst. Bacteriol..

[bib10] Fu D., Cui H., Zhang Y. (2018). Lack of ClC-2 alleviates high fat diet-induced insulin resistance and non-alcoholic fatty liver disease. Cell. Physiol. Biochem..

[bib11] Ge X., Wang C., Chen H., Liu T., Chen L., Huang Y., Zeng F., Liu B. (2020). Luteolin cooperated with metformin hydrochloride alleviates lipid metabolism disorders and optimizes intestinal flora compositions of high-fat diet mice. Food Funct..

[bib12] Geirnaert A., Steyaert A., Eeckhaut V., Debruyne B., Arends J., Immerseel F.V., Boon N., Wiele T.V. (2014). *Butyricicoccus pullicaecorum*, a butyrate producer with probiotic potential, is intrinsically tolerant to stomach and small intestine conditions. Anaerobe.

[bib13] Gerritsen J., Hornung B., Ritari J., Paulin L., Rijkers G.T., Schaap P.J., Vos W.M., Smidt H. (2019). A comparative and functional genomics analysis of the genus Romboutsia provides insight into adaptation to an intestinal lifestyle. bioRxiv.

[bib14] Ghaly S., Kaakoush N.O., Hart P.H. (2020). Effects of uvr exposure on the gut microbiota of mice and humans. Photochem. Photobiol. Sci..

[bib15] Guo W.L., Deng J.C., Pan Y.Y., Xu J.X., Hong J.L., Shi F.F., Liu G.L., Qian M., Bai W.D., Zhang W., Liu B., Zhang Y.Y., Luo P.J., Ni L., Rao P.F., Lv X.C. (2020). Hypoglycemic and hypolipidemic activities of *Grifola frondosa* polysaccharides and their relationships with the modulation of intestinal microflora in diabetic mice induced by high-fat diet and streptozotocin. Int. J. Biol. Macromol..

[bib16] He M., Shi B. (2017). Gut microbiota as a potential target of metabolic syndrome: the role of probiotics and prebiotics. Cell Biosci..

[bib17] He K., Hu Y., Ma H., Zou Z., Xiao Y., Yang Y. (2016). Rhizoma Coptidis alkaloids alleviate hyperlipidemia in B6 mice by modulating gut microbiota and bile acid pathways. Biochim. Biophys. Acta.

[bib18] Higashikawa F., Noda M., Awaya T., Danshiitsoodol N., Matoba Y., Kumagai T., Sugiyama M. (2016). Antiobesity effect of *Pediococcus pentosaceus* LP28 on overweight subjects: a randomized, double-blind, placebo-controlled clinical trial. Eur. J. Clin. Nutr..

[bib19] Hoyles L., Fernández-Real J.M., Federici M., Serino M., Abbott J., Charpentier J., Heymes C., Luque J.L., Anthony E., Barton R., Chilloux J., Myridakis A., Martinez-Gili L., Moreno-Navarrete J.M., Benhamed F., Azalbert V., Blasco-Baque V., Puig J., Xifra G., Ricart W., Tomlinson C., Woodbridge M., Cardellini M., Davato F., Cardolini I., Porzio O., Gentileschi P., Lopez F., Foufelle F., Butcher S., Holmes E., Nicholson J.K., Postic C., Burcelin R., Dumas M. (2018). Molecular phenomics and metagenomics of hepatic steatosis in non-diabetic obese women. Nat. Med..

[bib20] Huang Z.R., Chen M., Guo W.L., Li T.T., Liu B., Bai W.D., Ai L.Z., Rao P.F., Ni L., Lv X.C. (2020). Monascus purpureus-fermented common buckwheat protects against dyslipidemia and non-alcoholic fatty liver disease through the regulation of liver metabolome and intestinal microbiome. Food Res. Int..

[bib21] Hubbard B., Doege H., Punreddy S., Wu H., Huang X., Kaushik V.K., Mozell R.L., Byrnes J.J., Stricker-Krongrad A., Chou C.J., Tartaglia L.A., Lodish H.F., Stahl A., Gimeno R.E. (2006). Mice deleted for fatty acid transport protein 5 have defective bile acid conjugation and are protected from obesity. Gastroenterology.

[bib22] Irmler S., Bavan T., Oberli A., Roetschi A., Badertscher R., Guggenbühl B., Berthoud H. (2013). Catabolism of serine by *Pediococcus acidilactici* and *Pediococcus pentosaceus*. Appl. Environ. Microbiol..

[bib23] Ishikawa H., Ino S., Nakashima T., Matsuo H., Takahashi Y., Kohda C., Ōmura S., Iyoda M., Tanaka K. (2020). Oral administration of trehangelin-A alleviates metabolic disorders caused by a high-fat diet through improvement of lipid metabolism and restored beneficial microbiota. Obes. Res. Clin. Pract..

[bib24] Jain A.K., Kumar M., Ghosh M., Ganguli A. (2013). Modeling in vitro cholesterol reduction in relation to growth of probiotic *Lactobacillus casei*. Microbiol. Immunol..

[bib25] Jang W.J., Kim C.E., Jeon M.H., Lee S.J., Lee J.M., Lee E.W., Hasan M.T. (2021). Characterization of *Pediococcus acidilactici* FS2 isolated from Korean traditional fermented seafood and its blood cholesterol reduction effect in mice. J. Funct.Foods.

[bib26] Jaramillo-Torres A., Rawling M.D., Rodiles A., Mikalsen H.E., Johansen L.H., Tinsley J., Forberg T., Aasum E., Castex M., Merrifield D.L. (2019). Influence of dietary supplementation of probiotic *Pediococcus acidilactici* MA18/5M during the transition from freshwater to seawater on intestinal health and microbiota of Atlantic Salmon (Salmo salar L.). Front. Microbiol..

[bib27] Jiang T., Wu H., Yang X., Li Y., Zhang Z., Chen F., Zhao L., Zhang C. (2020). *Lactobacillus mucosae* strain promoted by a high-fiber diet in genetic obese child alleviates lipid metabolism and modifies gut microbiota in apoe-/- mice on a western diet. Microorganisms.

[bib28] Kim W.S., Lee J.Y., Singh B., Maharjan S., Hong L., Lee S.M., Cui L.H., Lee K.J., Kim G., Yun C.H., Kang S.K., Choi Y.J., Cho C.S. (2018). A new way of producing pediocin in *Pediococcus acidilactici* through intracellular stimulation by internalized inulin nanoparticles. Sci. Rep..

[bib29] Kim S.H., Wang J.K., Kang S.S. (2019). Inhibitory effect of bacteriocin-producing *Lactobacillus brevis* DF01 and *Pediococcus acidilactici* K10 isolated from kimchi on enteropathogenic bacterial adhesion. Food Biosci..

[bib30] Lanjekar V.B., Marathe N.P., Shouche Y.S., Ranade D.R. (2015). *Clostridium punense* sp. nov., an obligate anaerobe isolated from healthy human faeces. Int. J. Syst. Evol. Microbiol..

[bib31] Li T., Owsley E., Matozel M., Hsu P., Novak C.M., Chiang J. (2010). Transgenic expression of cholesterol 7α‐hydroxylase in the liver prevents high‐fat diet–induced obesity and insulin resistance in mice. Hepatology.

[bib32] Li S., Qi C., Zhu H., Yu R., Xie C., Peng Y., Yin S.W., Fan J., Zhao S., Sun J. (2019). *Lactobacillus reuteri* improves gut barrier function and affects diurnal variation of the gut microbiota in mice fed a high-fat diet. Food Funct..

[bib33] Li T.T., Huang Z.R., Jia R.B., Lv X.C., Zhao C., Liu B. (2021). Spirulina platensis polysaccharides attenuate lipid and carbohydrate metabolism disorder in high-sucrose and high-fat diet-fed rats in association with intestinal microbiota. Food Res. Int..

[bib34] Liu Y., Song X., Zhou H., Xue Z., Xia Y., Dong X., Wei Z., Tang S., Wang L., Wen S. (2018). Gut microbiome associates with lipid-lowering effect of rosuvastatin in vivo. Front. Microbiol..

[bib35] Lv X.C., Guo W.L., Li L., Yu X.D., Liu B. (2019). Polysaccharide peptides from *Ganoderma lucidum* ameliorate lipid metabolic disorders and gut microbiota dysbiosis in high-fat diet-fed rats. J. Funct.Foods.

[bib36] Lv X.C., Chen M., Huang Z.R., Guo W.L., Ai L.Z., Bai W.D., Yu X.D., Liu Y.L., Rao P.F., Ni L. (2021). Potential mechanisms underlying the ameliorative effect of *Lactobacillus paracasei* FZU103 on the lipid metabolism in hyperlipidemic mice fed a high-fat diet. Food Res. Int..

[bib37] Ma Z., Chu L., Liu H., Wang W., Li J., Yao W., Yi J., Gao Y. (2017). Beneficial effects of paeoniflorin on non-alcoholic fatty liver disease induced by high-fat diet in rats. Sci. Rep..

[bib38] Mao S., Zhang R., Wang D., Zhu W. (2012). The diversity of the fecal bacterial community and its relationship with the concentration of volatile fatty acids in the feces during subacute rumen acidosis in dairy cows. BMC Vet. Res..

[bib39] Martinez-Guryn K., Hubert N., Frazier K., Urlass S., Musch M.W., Ojeda P., Pierre J.F., Miyoshi J., Sontag T.J., Cham C.M., Reardon C.A., Leone V., Chang E.B. (2018). Small intestine microbiota regulate host digestive and absorptive adaptive responses to dietary lipids. Cell Host Microbe.

[bib40] Meikle P., Summers S. (2017). Sphingolipids and phospholipids in insulin resistance and related metabolic disorders. Nat. Rev. Endocrinol..

[bib41] Moon Y.J., Baik S.H., Cha Y.S. (2014). Lipid-lowering effects of *Pediococcus acidilactici* M76 isolated from Korean traditional makgeolli in high fat diet-induced obese mice. Nutrients.

[bib42] Morris M.J., Beilharz J.E., Maniam J., Reichelt A.C., Westbrook R.F. (2015). Why is obesity such a problem in the 21st century? the intersection of palatable food, cues and reward pathways, stress, and cognition. Neurosci. Biobehav. Rev..

[bib43] Nergiz-Unal R., Ulug E., Kisioglu B., Tamer F., Yuruk A.A. (2020). Hepatic cholesterol synthesis and lipoprotein levels impaired by dietary fructose and saturated fatty acids in mice: insight on PCSK9 and CD36. Nutrition.

[bib44] Oh Y.J., Kim H.J., Kim T.S., Yeo I.H., Ji G.E. (2019). Effects of *Lactobacillus plantarum* PMO08 alone and combined with chia seeds on metabolic syndrome and parameters related to gut health in high-fat diet-induced obese mice. J. Med. Food.

[bib45] Ooi L.G., Liong M.T. (2010). Cholesterol-lowering effects of probiotics and prebiotics: a review of in vivo and in vitro findings. Int. J. Mol. Sci..

[bib46] Peters A., Krumbholz P., Jger E., Heintz-Buschart A., Stubert C. (2019). Metabolites of lactic acid bacteria present in fermented foods are highly potent agonists of human hydroxycarboxylic acid receptor 3. PLoS Genet..

[bib47] Qu W., Yuan X., Zhao J., Zhang Y., Hu J., Wang J. (2017). Dietary advanced glycation end products modify gut microbial composition and partially increase colon permeability in rats. Mol. Nutr. Food Res..

[bib48] Ren Q., Liu X.Q., Zhou X.W., Zhou X., Li X.T. (2021). Effects of Huatan Jiangzhuo decoction on diet-induced hyperlipidemia and gene expressions in rats. Chin. J. Nat. Med..

[bib49] Russell J.P., Mohammadi E., Ligon C.O., Johnson A.C., Gershon M.D., Rao M., Shen Y., Chan C.C., Eidam H.S., DeMartino M.P., Cheung M., Oliff A.I., Kumar S., Greenwood-Van Meerveld B. (2019). Exploring the potential of ret kinase inhibition for irritable bowel syndrome: a preclinical investigation in rodent models of colonic hypersensitivity. J. Pharmacol. Exp. Therapeut..

[bib50] Saadat Y.R., Khosroushahi A.Y., Gargari B.P. (2019). A comprehensive review of anticancer, immunomodulatory and health beneficial effects of the lactic acid bacteria exopolysaccharides. Carbohydr. Polym..

[bib51] Shang Q., Song G., Zhang M., Shi J., Xu C., Hao J., Li G., Yu G. (2017). Dietary fucoidan improves metabolic syndrome in association with increased akkermansia population in the gut microbiota of high-fat diet-fed mice. J. Funct.Foods.

[bib52] Shao Y., Huo D., Peng Q., Pan Y., Jiang S., Liu B., Zhang J.C. (2017). *Lactobacillus plantarum* HNU082-derived improvements in the intestinal microbiome prevent the development of hyperlipidaemia. Food Funct..

[bib53] Shearer J., Duggan G., Weljie A., Hittel D.S., Wasserman D.H., Vogel H.J. (2008). Metabolomic profiling of dietary-induced insulin resistance in the high fat-fed C57BL/6J mouse. Diabetes Obes. Metabol..

[bib54] Si X., Shang W., Zhou Z., Shui G., Lam S.M., Blanchard C., Strappe P. (2018). Gamma-aminobutyric acid enriched rice bran diet attenuates insulin resistance and balances energy expenditure via modification of gut microbiota and short-chain fatty acids. J. Agric. Food Chem..

[bib55] Suleiman J.B., Nna V.U., Zakaria Z., Othman Z.A., Mohamed M. (2020). Obesity-induced testicular oxidative stress, inflammation and apoptosis: protective and therapeutic effects of orlistat. Reprod. Toxicol..

[bib56] Sultana H., Komai M., Shirakawa H. (2021). The role of vitamin K in cholestatic liver disease. Nutrients.

[bib57] Takahashi N., Mori Y. (2011). Trp channels as sensors and signal integrators of redox status changes. Front. Pharmacol..

[bib58] Tang Y., Zhang J., Li J., Lei X., Xu D., Wang Y., Li C., Li X., Mao Y. (2019). Turnover of bile acids in liver, serum and caecal content by high-fat diet feeding affects hepatic steatosis in rats. BBA-Mol. Cell Biol. L..

[bib59] Uzun M.A., Koksal N., Kadioglu H., Gunerhan Y., Aktas S., Dursun N., Sehirli A.O. (2009). Effects of n-acetylcysteine on regeneration following partial hepatectomy in rats with nonalcoholic fatty liver disease. Surg. Today.

[bib60] Villanueva-Millán M.J., Pérez-Matute P., Oteo J.A. (2015). Gut microbiota: a key player in health and disease. A review focused on obesity. J. Physiol. Biochem..

[bib61] Wan Y., Tong W., Zhou R., Li J., Yuan J., Wang F., Li D. (2019). Habitual animal fat consumption in shaping gut microbiota and microbial metabolites. Food Funct..

[bib62] Wang J., Zou Y., Huang C., Lu C., Zhang L. (2012). Protective effects of tiopronin against high fat diet-induced non-alcoholic steatohepatitis in rats. Acta Pharmacol. Sin..

[bib63] Wang T., Zhou Z.X., Sun L.X., Li X., Xu Z., Chen M., Zhao G., Jiang Z., Zhang L. (2014). Resveratrol effectively attenuates α-naphthyl-isothiocyanate-induced acute cholestasis and liver injury through choleretic and anti-inflammatory mechanisms. Acta Pharmacol. Sin..

[bib64] Wang C., Wang H., Zhao Z., Xiao S., Wang J. (2019). *Pediococcus acidilactici* AS185 attenuates early atherosclerosis development through inhibition of lipid regulation and inflammation in rats. J. Funct.Foods.

[bib65] Wang Y., You Y., Tian Y., Sun H., Liu J. (2020). *Pediococcus pentosaceus* PP04 ameliorates high-fat diet-induced hyperlipidemia by regulating lipid metabolism in C57BL/6N mice. J. Agric. Food Chem..

[bib66] Wei W., Jiang W., Tian Z., Wu H., Ning H., Yan G., Zhang Z., Li Z., Dong F., Sun Y., Li Y., Han T., Wang M., Sun C., Fecal G. (2021). *Streptococcus* and g. *Eubacterium_coprostanoligenes*_group combined with sphingosine to modulate the serum dyslipidemia in high-fat diet mice. Clin. Nutr..

[bib67] Wuyts S., Beeck W.V., Allonsius C.N., Broek M., Lebeer S. (2020). Applications of plant-based fermented foods and their microbes. Curr. Opin. Biotechnol..

[bib68] Xie M., Chen G., Wan P., Dai Z., Hu B., Chen L., Ou S., Zeng X., Sun Y. (2017). Modulating effects of dicaffeoylquinic acids from ilex kudingcha on intestinal microecology in vitro. J. Agric. Food Chem..

[bib69] Yamasaki M., Minesaki M., Iwakiri A., Miyamoto Y., Ogawa K., Nishiyama K., Tsend-Ayush C., Oyunsuren T., Li Y., Nakano T., Takeshita M., Arima Y. (2020). Lactobacillus plantarum 06CC2 reduces hepatic cholesterol levels and modulates bile acid deconjugation in Balb/c mice fed a high-cholesterol diet. Food Sci. Nutr..

[bib70] Yang G., Hong E., Oh S., Kim E. (2020). Non-viable *Lactobacillus johnsonii* JNU3402 protects against diet-induced obesity. Foods.

[bib71] Yeon-Jeong M., Sang-Ho B., Youn-Soo C. (2014). Lipid-lowering effects of *Pediococcus acidilactici* M76 isolated from Korean traditional makgeolli in high fat diet-induced obese mice. Nutrients.

[bib72] Yoo J.Y., Kim S.S. (2016). Probiotics and prebiotics: present status and future perspectives on metabolic disorders. Nutrients.

[bib73] Zhang Q., Wang G.J., Wu D., Zhu L.L., Ma B., Du Y. (2009). Application of GC/MS-based metabonomic profiling in studying the lipid-regulating effects of Ginkgo biloba extract on diet-induced hyperlipidemia in rats. Acta Pharmacol. Sin..

[bib74] Zhang X., Zhao Y., Xu J., Xue Z., Zhang M., Pang X., Zhang X., Zhao L. (2015). Modulation of gut microbiota by berberine and metformin during the treatment of high-fat diet-induced obesity in rats. Sci. Rep..

[bib75] Zhang Q., Fan X., Ye R., Hu Y., Zheng T., Shi R., Cheng W., Lv X., Chen L., Liang P. (2020). The effect of simvastatin on gut microbiota and lipid metabolism in hyperlipidemic rats induced by a high-fat diet. Front. Pharmacol..

[bib76] Zhang X., Zhan Y., Lin W., Zhao F., Guo C., Chen Y., Du M., Li D., Zhang L., An W., Wang H.R., Xie P. (2020). Smurf1 aggravates non-alcoholic fatty liver disease by stabilizing SREBP-1c in an E3 activity-independent manner. Faseb. J..

[bib77] Zheng S., Shao S., Qiao Z., Chen X., Piao C., Yu Y., Gao F., Zhang J., Du J. (2017). Clinical parameters and gut microbiome changes before and after surgery in thoracicaortic dissection in patients with gastrointestinal complications. Sci. Rep..

[bib78] Zheng B., Wang T., Wang H., Chen L., Zhou Z. (2020). Studies on nutritional intervention of rice starch- oleic acid complex (resistant starch type V) in rats fed by high-fat diet. Carbohydr. Polym..

[bib79] Zhong Y., Nyman M., Fåk F. (2015). Modulation of gut microbiota in rats fed high-fat diets by processing whole-grain barley to barley malt. Mol. Nutr. Food Res..

[bib80] Zhu L., Fu J., Xiao X., Wang F., Jin M., Fang W., Wang Y., Zong X. (2021). Faecal microbiota transplantation-mediated jejunal microbiota changes halt high-fat diet-induced obesity in mice via retarding intestinal fat absorption. Microb. Biotechnol..

