# Supplementary material for: Pediococcus acidilactici FZU106 alleviates high-fat diet-induced lipid metabolism disorder in association with the modulation of intestinal microbiota in hyperlipidemic rats
Source: Curr Res Food Sci. 2022 Apr 27;5:775–88. doi: 10.1016/j.crfs.2022.04.009 (PMC9064835; doi:10.1016/j.crfs.2022.04.009)
Supplement: Multimedia component 1 [file mmc1.doc]

**Supplementary materials**

**Table S1. Effect of bile salt concentration on the viability of different strains of lactic acid bacteria from the traditional brewing process of** ***Hongqu* rice wine.**

| **Bacterial isolates** | **Bile salt concentration** | | | |
| --- | --- | --- | --- | --- |
|  | **0%** | **0.3%** | **0.5%** | **1%** |
| *Leuconostoc mesenteroides* FZU123 | 43.6 ± 0.50 | 25.4 ± 0.45 | 10.4 ± 0.24 | 2.6 ± 0.44 |
| *Pediococcus pentosaceus* FZU003 | 42.0 ± 0.58 | 23.4 ± 0.36 | 13.4 ± 0.28 | ND |
| *Lactobacillus plantarum* FZU3013 | 43.3 ± 0.32 | 27.4 ± 0.38 | 16.4 ± 0.56 | 6.8 ± 0.18 |
| *Lactobacillus paracasei* FZU103 | 42.8 ± 0.55 | 28.4 ± 0.33 | 16.8 ± 0.28 | 8.0 ± 0.16 |
| *Lactobacillus brevis* FZU006 | 43.8 ± 0.34 | 23.4 ± 0.55 | 9.4 ± 0.10 | 1.5 ± 0.38 |
| *Lactococcus lactis subsp. cremoris* FZU203 | 43.3 ± 0.20 | 26.5 ± 0.14 | 13.8 ± 0.25 | 3.2 ± 0.10 |
| ***Pediococcus acidilactici* FZU106** | **43.1 ± 0.32** | **29.8 ± 0.18** | **17.2 ± 0.30** | **8.6 ± 0.25** |
| *Lactobacillus coryniformis* FZU606 | 42.5 ± 0.38 | 20.4 ± 0.18 | 6.2 ± 0.30 | ND |
| *Lactococcus lactis* subsp. lactis FZU369 | 42.8 ± 0.48 | 22.4 ± 0.42 | 8.8 ± 0.36 | ND |

CFU, colony forming unit after 24 h incubation at 37 ± 2°C under anaerobic condition. Values are represented as mean ± standard deviation (*n* = 3). ND, not detectable with the indicators employed in this study.

**Experimental method of resistance to bile salts:** Growth rate of bacterial cultures in MRS broth containing different levels (0%, 0.3%, 0.5% and 1%) of bile salts were determined. Freshly prepared cultures (8 log10 CFU/mL) were inoculated (1%) into medium and incubated at 37°C for 24 h under anaerobic condition. 1 mL of sample was taken after treatment for 5 h, and serial dilutions were made using peptone water diluents. Samples were plated onto MRS agar, and the plates were incubated at 37°C for 48 h in an anaerobic jar.

**Result description:** The strains were screened for their ability to tolerate the bile salt. Table S1 shows the effect of bile salts on the growth of the strains. Significant variations existed among the cultures with regard to their ability to grow in MRS broth supplemented with bile salts (P < 0.05). *Lactobacillus coryniformis* FZU606 and *Lactococcus lactis* subsp. lactis FZU369 were found to be the least bile tolerant, while *Pediococcus acidilactici* FZU106 was found to be the most resistant. In a comparison of all the other strains, *Lactobacillus coryniformis* FZU606 and *Lactococcus lactis* subsp. lactis FZU369 were more sensitive to bile salts, with significantly lower growth rates in the medium with 0.5% (w/v) bile acids than in the absence of bile (P < 0.05). *Pediococcus pentosaceus* FZU003, *Lactobacillus coryniformis* FZU606 and *Lactococcus lactis* subsp. lactis FZU369 show no growth at 1% bile after 5 h. *Pediococcus acidilactici* FZU106 is the most bile resistant strain, it shows viability (8.6 ± 0.25%) at 1% bile concentration after 5 h.

**Table S2.** In vitro cholesterol assimilation by different strains of lactic acid bacteria from the traditional brewing process of *Hongqu* rice wine.

| **Bacterial isolates** | **Cholesterol concentration** | | | |
| --- | --- | --- | --- | --- |
|  | **50 μg/ml** | **100 μg/ml** | **150 μg/ml** | **200 μg/ml** |
| *Leuconostoc mesenteroides* FZU123 | 18.22 ± 0.68 | 19.04 ± 0.23 | 22.15 ± 0.48 | 20.40 ± 0.28 |
| *Pediococcus pentosaceus* FZU003 | 15.08 ± 0.50 | 17.80 ± 0.45 | 18.36 ± 0.50 | 17.10 ± 0.34 |
| *Lactobacillus plantarum* FZU3013 | 22.04± 1.04 | 22.34 ± 0.28 | 24.46 ± 0.46 | 25.20 ± 0.38 |
| *Lactobacillus paracasei* FZU103 | 26.55 ± 0.60 | 27.30 ± 0.60 | 28.25 ± 0.77 | 27.24 ± 0.08 |
| *Lactobacillus brevis* FZU006 | 19.67 ± 0.45 | 20.23 ± 0.18 | 21.80 ± 0.28 | 19.88 ± 0.40 |
| *Lactococcus lactis subsp. cremoris* FZU203 | 16.45 ± 0.26 | 19.34 ± 0.15 | 21.66 ± 0.35 | 24.56 ± 0.18 |
| ***Pediococcus acidilactici* FZU106** | **28.42 ± 0.32** | **31.06 ± 0.45** | **30.86 ± 0.42** | **29.68 ± 0.14** |
| *Lactobacillus coryniformis* FZU606 | 15.24 ± 0.60 | 16.80 ± 0.18 | 18.80 ± 0.28 | 18.20 ± 0.42 |
| *Lactococcus lactis* subsp. lactis FZU369 | 16.08 ± 0.40 | 18.04 ± 0.28 | 20.18 ± 0.60 | 19.10 ± 0.26 |

Cholesterol assimilation is defined as cholesterol removal per ml of culture broth after 24 h of incubation at 37 ± 2°C under anaerobic condition. Results are expressed as mean ± standard deviation; n = 3.

**Experimental method of cholesterol reduction assay:** In vitro cholesterol reduction was determined by growing cells at 37°C in MRS broth. Water soluble cholesterol (polyoxyethanyl-cholesteryl sebacate, Sigma) was filter sterilized and added to the broth at a final concentration of 50–200 μg/mL, inoculated with each strain at 1% level and incubated anaerobically at 37°C for 24 h. Following incubation the cells were harvested by centrifuging (10,000 ×*g*, 4°C, 10 min) and the supernatant was collected. The cholesterol content was determined using a modified colorimetric method as described by Rudel and Morris and Gilliland et al.. FeCl_3_ work solution (1.5 ml) was added to the tube containing sample, after through mixing the solution was allowed to stand for 10 min. Then 1 ml of concentrated sulphuric acid (H_2_SO_4_) was added. The solutions were placed in the dark for 45 min and absorbance was read at 560 nm (Spectronic 20D, Thermo Scientific, USA) after 24 h. The activity of cholesterol lowering (μg/ml culture broth) was calculated as follows: Cholesterol assimilation (μg/ml) = (C1−C2) or (C1−C2)/(W2−W1), where C1 and C2 represent the cholesterol concentration of the uninoculated and inoculated medium, respectively, and W1 and W2 represent the weight of culture per milliliter of medium before and after the incubation period.

**Results:** The amount of cholesterol assimilated by the different strains showed wide variation after 24 h of anaerobic growth. In general, *Pediococcus acidilactici* FZU106 strain exhibited high cholesterol assimilation compared to other strain. *Pediococcus acidilactici* FZU106 strain assimilate cholesterol about 29.68 ± 0.14 μg/mL in media supplemented with 200 μg/mL cholesterol. Cholesterol assimilation (μg/mL) from MRS broth was significantly high (*P* ≤ 0.05) for strains *Pediococcus acidilactici* FZU106 and *Lactobacillus paracasei* FZU103 without bile salts, whilst the lowest cholesterol removal values were recorded for *Pediococcus pentosaceus* FZU003 and *Lactobacillus coryniformis* FZU606, respectively.

**References:**

Rudel, L. L. and Morris, M. D.: Determination of cholesterol using o-phthalaldehyde, J. Lipid Res., 14, 364-366 (1973).

Gilliland, S. E., Nelson, C. R., and Maxwell, C.: Assimilation of cholesterol by Lactobacillus acidophilus, Appl. Environ. Microbiol., 49, 377-381 (1985).

**Fig. S1.** Correlation network between the biochemical parameters and the key microbial phylotypes. The edge width and colour (pink: positive and green: negative) are proportional to the correlation strength. Only the significant edges were drawn in the network (|r| > 0.6, FDR adjusted *p < 0.05,**p < 0.01).
